# Role of Old Antibiotics in the Era of Antibiotic Resistance. Highlighted Nitrofurantoin for the Treatment of Lower Urinary Tract Infections

**DOI:** 10.3390/antibiotics3010039

**Published:** 2014-02-10

**Authors:** Maria Jose Munoz-Davila

**Affiliations:** Microbiology laboratory, Rafael Méndez Hospital, Ctra. Nacional 340, Km. 589, Lorca, Murcia 30817, Spain; E-Mail: mariajose.munoz5@um.es; Tel.: +34-968-44-50-00; Fax: +34-968-44-24-212

**Keywords:** nitrofurantoin, urinary tract infection, therapeutic use

## Abstract

Bacterial infections caused by antibiotic-resistant isolates have become a major health problem in recent years, since they are very difficult to treat, leading to an increase in morbidity and mortality. Nitrofurantoin is a broad-spectrum bactericidal antibiotic that, through a complex mode of action which is not completely understood, affects both Gram-negative and Gram-positive bacteria. Nitrofurantoin has been used successfully for a long time for the prophylaxis and treatment of acute lower urinary tract infections in adults, children and pregnant women, but the increased emergence of antibiotic resistance has made nitrofurantoin a suitable candidate for the treatment of infections caused by multidrug-resistant pathogens. Here, we review the mechanism of action, antimicrobial spectrum, pharmacology and safety profile of nitrofurantoin. We also investigate the therapeutic use of nitrofurantoin, including recent data which highlight its role in the management of community urinary tract infection, especially in cases of multidrug-resistant isolates, in which oral active antimicrobials are limited resources nowadays.

## 1. Introduction

**Figure 1:**
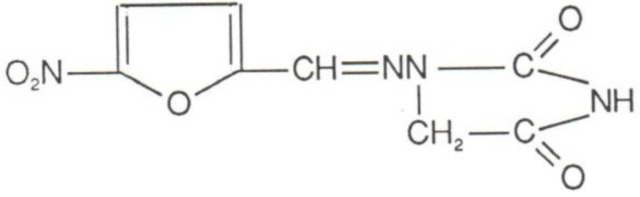
Chemical structure of nitrofurantoin.

Nitrofurantoin, a chemotherapeutic compound of the nitrofuran family, was introduced into clinical practice in 1952. Nitrofurantoin is a synthetic antimicrobial derived from furan by the addition of a nitro group and a side chain containing hydantoin (Figure 1). Nitrofurantoin is a weak acid and its solubility is affected by pH [1].

## 2. Mechanism of Action

The exact mode of action of nitrofurantoin is not completely understood, though it is mainly known to inhibit a number of bacterial enzymes that participate in bacterial carbohydrate metabolism at three points in the Krebs cycle [1] as well as interfering with cell wall synthesis [2]. The nitrogroup coupled onto the heterocyclic furan ring represents the specific active site of the drug and has to be activated by microbial nitroreductases [3].

## 3. Antimicrobial Spectrum

Nitrofurantoin spectrum of *in vitro* susceptibility includes the majority of *Escherichia coli*, *Citrobacter* species, group B streptococci, enterococci, *Staphylococcus aureus*, *S. epidermidis*, *Klebsiella pneumoniae* and *Enterobacter* species [1]. Thus, its antibacterial spectrum is broad and is particularly effective against the main uropathogens, hence its use for the treatment of urinary tract infections (UTI). Resistance to this drug has remained virtually unchanged since its discovery [4].

## 4. Pharmacology

Nowadays, in Spain, the compound is marketed as both an oral suspension and tablets. The bioavailability is about 90% and the urinary excretion is 40% [5].

### 4.1. Absorption

Nitrofurantoin is well absorbed from the gastrointestinal tract with the most absorption occurring in the proximal small bowel. There are many factors which affect bioavailability, but studies have shown that the amount of drug absorbed and the duration of therapeutic urinary concentrations are substantially increased if nitrofurantoin is taken with food [2]. Particle size also affects bioavailability. Thus, the macrocristalline form is more slowly absorbed in the GI tract and is excreted more slowly in the urine than the microcrystalline formulation. This decreased rate of absorption significantly decreases the incidence of nausea and vomiting associated with the microcrystalline form [1]. Due to the effective gastro-intestinal absorption of nitrofurantoin, the effect on the intestinal flora is minimal [6].

### 4.2. Distribution and Excretion

Nitrofurantoin is excreted almost exclusively in the urine and bile. Urinary excretion results from glomerular filtration, tubular secretion and tubular reabsorption. Tubular reabsorption of nitrofurantoin is pH dependent. Thus, manipulating the urinary pH permits preferential concentration of nitrofurantoin into the upper or lower urinary tract [7]. Nitrofurantoin does not penetrate aqueous humor, cerebrospinal fluid, prostatic secretions, amniotic fluid or umbilical cord serum in therapeutic concentrations when administered parenterally to man or animals [1]. Oral administration to breast feeding mothers results in minimal drug concentrations in breast milk [8,9].

### 4.3. Excretion in Patients with Impaired Renal Function

The excretion of the drug is directly related to creatinine clearance [10]. In the presence of impaired renal function, the urine levels fall below the therapeutic range while the serum levels increase into the toxic range. Thus, its efficacy is limited in the setting of renal impairment, with an associated greater risk of toxic effects and adverse reactions [11].

### 4.4. Interactions

It has been suggested that antacid therapy would increase the ionization of nitrofurantoin, resulting in decreased absorption. Also, nitrofurantoin is a potent inhibitor of primary adenine diphosphate-induced platelet aggregation *in vitro*. Nitrofurantoin can alter a number of laboratory test results. Thus, there may be elevation of the urinary creatinine value. Also, the urine glucose determination using Benedict´s qualitative reagent may yield a false-positive result. Serum levels of glucose, bilirubin, alkaline phosphatase and blood urea nitrogen may be spuriously elevated. Also, patients should be warned that the color of the urine may be altered (brown) [1].

## 5. Safety Profile

Nitrofurantoin is, overall, a relatively safe drug. The overall experience after more than three decades of extensive use shows a very low reported side-effect incidence of less than 0.001 per cent based on total courses of therapy [12,13,14]. However, adverse reactions described, mainly related to long-term usage, have included gastrointestinal disturbances, skin eruptions, hematologic disorders, neurological defects, hepatotoxicity, pulmonary complications and miscellaneous abnormalities. A brief summary of the main adverse reactions is described next.

Gastrointestinal disturbances (anorexia, nausea, vomiting) are the most common side effects. They usually develop during the first week of therapy and efforts have been made to reduce their frequency by altering the nitrofurantoin crystal size, thus modifying the absorption. Skin eruption, consisting of macular, maculopapular or urticarial lesions, are the second most common side effect of nitrofurantoin. Hemolytic anemia in patients with red blood cells deficient in the enzyme glucose-6-phosphate dehydrogenase (G6PD) is a well-documented hematologic complication of nitrofurantoin therapy [2]. A serious adverse reaction to nitrofurantoin described is pheripheral neuropathy [15]. Nitrofurantoin-induced hepatotoxicity is a rare event that usually is readily reversible with discontinuation of the medication. However, nitrofurantoin-induced pulmonary reactions have developed in hundreds of patients. This untoward reaction has been classified arbitrarily into acute, subacute, and chronic forms. The classical acute pulmonary reaction syndrome is characterized by the sudden onset of fever, chills, cough, myalgia and dyspnea. This reaction develops within hours to weeks of the ingestion of the drug. Subacute pulmonary reactions from nitrofurantoin usually develop after one month of drug exposure and are characterized by persistent and progressive cough, dyspnea, orthopnea and fever. The chronic nitrofurantoin pulmonary reaction is associated with the insidious development of non-productive cough and dyspnea. Current guidelines and primary care prescribing systems should emphasize the potential for pulmonary toxicity, which is reversible in case of early recognition [16].

The incidence of these side effects is difficult to ascertain, and they are probably on the same order of magnitude (or less) than severe antibiotic associated diarrhoea caused by beta-lactam drugs or fluoroquinolones, or severe skin eruptions caused by trimethoprim/sulfamethoxazole [15].

While medical literature generally defines nitrofurantoin as an antibiotic that is safe for use during the first trimester of pregnancy, new concerns about a possible association between congenital malformations following exposure to nitrofurantoin during the first trimester of pregnancy have recently surfaced. During the last decade, several studies have suggested an increased risk of enophthalmia, cardiovascular malformations, oral cleft, and scull anomalies [17,18]. However, far more studies have suggested that nitrofurantoin is not associated with increased teratogenic risk [19,20,21,22,23,24]. Also, Goldberg *et al*. [25] support its use during the first trimester of pregnancy to treat UTI by failing to detect teratogenic risks in a large cohort of exposed women. As far as we know, nitrofurantoin does not cross the placenta [26]. Moreover, overall, it does not appear to exert any untoward effects on the fetus when administered to the pregnant female, except in the latter stages [27]. However, for most outpatient procedures, beta-lactam antibiotics are preferred for the treatment of urinary tract infections in pregnant women.

## 6. Therapeutic Use

### 6.1. Classical

The conventional dosage of nitrofurantoin for an established urinary tract infection is 50 mg or 100 mg four times a day and it started being prescribed both in adults and children for the treatment of acute symptomatic urinary tract infections, also for the treatment of recurrent urinary tract infections and finally for the prophylaxis of recurrent urinary tract infections [1]. Due to its inability to achieve therapeutic blood concentrations, this compound has been relegated to a position of secondary importance. Thus, nitrofurantoin should never be administered to patients with acute bacterial pyelonephritis (as this disease can be accompanied by bacteremia) or to men with recurrent urinary tract infections, as these infections are related with prostatitis and nitrofurantoin does not penetrate tissues well [2].

Another of its main indications is that of bacteriuria of pregnancy, which occurs in approximately 7 to 10 per cent of all pregnant women [28]. Nitrofurantoin is also recommended for the treatment of catheter-associated bacteriuria, which is the most common cause of urinary tract infection. In this sense, nitrofurantoin is used prophylactically during or following urinary tract instrumentation. Nitrofurantoin has been found to prevent bacteriuria in patients with neurogenic bladders using self-catheterization and after intermittent catheterization in patients in the areflexic bladder phase after spinal cord injury [29].

Duration of nitrofurantoin therapy has always remained controversial. Lumbiganon and co-workers sought to find out if the dosing schedule of nitrofurantoin could be decreased from the traditional seven days of treatment to one day to increase compliance, while retaining its efficacy in the treatment of asymptomatic bacteriuria in pregnant women [30]. Although there were no significant differences in symptomatic infections, preterm deliveries and tolerance of subjects were observed between the short and long dosing schedules. More treatment failures, however, were seen in the short-dosing schedule, suggesting the superiority of the traditional-dosing schedule [31].

Also, a decrease in the number of doses per day has always been desirable. Recent data indicate that nitrofurantoin three times per day, instead of four, either in adults or in children, could be effective in the management of UTIs, increasing adherence to nitrofurantoin treatment and sparing the use of other antibiotics [32].

Recent data suggest nitrofurantoin as the first drug of choice for treating uncomplicated UTI in women. Nitrofurantoin has been recently compared to trimethoprim/sulfamethoxazole and proved equally effective. Moreover, it was less likely to cause a rash while having similar rates for any adverse event. Based on these findings nitrofurantoin should probably be considered the first drug of choice for treating uncomplicated UTI in women [33]. Also, Gupta *et al.* [34] reported that a 100 mg bid dosage of nitrofurantoin macrocrystal for 5 days had as good results as classical cotrimoxazole for 3 days for the treatment of acute uncomplicated cystitis in women. Regarding the prevention of recurrent UTI during pregnancy, no significant differences have been found between a combination of suppressive therapy with a daily dose of nitrofurantoin and close surveillance and close surveillance alone. Only sub-analyses in women with more than 90% follow-up show a decreased incidence of asymptomatic bacteriuria in women who received nitrofurantoin and close surveillance compared with close surveillance only [35]. Finally, one study of long-term antibiotics for preventing recurrent urinary tract infection in children demonstrated that, although nitrofurantoin was more effective than trimethoprim or cotrimoxazole in preventing repeat symptomatic infection or repeat positive urine culture, it was associated with a greater number of side effects, especially in children less than one month of age. The harmful effects of nitrofurantoin outweigh the prophylactic benefit and suggest that nitrofurantoin may not be an acceptable therapy. Patient compliance would be an important factor to consider in deciding on the use of nitrofurantoin as prophylaxis [36].

### 6.2. New Applications

Nowadays, nitrofurantoin is a synthetic nitrofuran antimicrobial agent that has been used for more than 50 years. It still has a role and continues to be prescribed, particularly in the ambulatory setting for uncomplicated UTIs, especially in its macro-crystalline formulation. In recent years, there has been a new interest in “rediscovering” new applications for older antibiotics due to changes in pathogen distribution and resistance. Regarding nitrofurantoin, it has been suggested for the treatment of UTI caused by multiresistant strains.

In this sense, nitrofurantoin is being used increasingly at present to treat vancomycin-resistant enterococci (VRE) nosocomial urinary tract infections (*i.e.*, catheter-associated bacteria). It is one of the few non-ampicillin derivatives that is active against enterococci. Nitrofurantoin is active against both vancomycin-sensitive enterococci (VSE) and VRE. It is the preferred oral antibiotic for nosocomial VSE or VRE catheter-associated bacteriuria [26].

Also, the progressive increase of extended-spectrum b-lactamase (ESBL) producing enteric bacteria in recent years has called for a re-evaluation of current antibiotic therapy for these infections. The rate of resistance to nitrofurantoin in recent surveys in the USA and Canada was 1.1% among 1,142 isolates of *E. coli* from outpatient urinary isolates [37]. Very similar results were found in France where 1.8% of all urinary *E. coli* isolates were resistant to nitrofurantoin in 2005 [38]. However, among 115 clinical isolates of *E. coli* ESBL producers, only 71.3% were sensitive to nitrofurantoin [39]. Also, *E. coli* resistance to nitrofurantoin has been reported to be high in a recent survey in Latin American hospitals [40] and in Italy [41]. In *K. pneumoniae*, the ESBL producers had significantly diminished susceptibility, as compared with a non-ESBL producer, to nitrofurantoin (*p* < 0.001) [42]. Because responses to nitrofurantoin may be less satisfactory and may require longer courses of therapy, nitrofurantoin is considered to be an alternative, rather than a first-line, therapeutic agent for this clinical syndrome [43]. These results are confirmed by Tasbakan *et al*. [44] who studied a total of 75 patients with lower urinary tract infection caused by culture-proven ESBL-producing nitrofurantoin-sensitive *E. coli* in the urine (>10^5^ CFU/mm^3^). Microbiological success was defined as a sterile control urine culture and it was achieved in 51 out of 75 patients (68%). Also, Chen *et al*. conclude that nitrofurantoin may be an alternative in the treatment of ESBL-producing *E. coli*-related lower UTI [43,44,45]. 

Nevertheless, activity against *E. coli* non-ESBL is excellent. In a recent study, the susceptibility for *E. coli* was 99.5%. High susceptibility of *E. coli* clinical isolates to nitrofurantoin (2.3% resistance rate), compared to trimethoprim/sulfamethoxazole (29%) or ciprofloxacin (24.2%) was recently confirmed [46]. However, nitrofurantoin is less susceptible against Gram-negative pathogens other than *E. coli*, such as *Klebsiella* spp. (69.2%) or *Enterobacter* spp. (63%). There is no activity against *Proteus* spp. or *P. aeruginosa* [47]. Nowadays, in uncomplicated cystitis, antibiotics exclusively reserved for this indication are preferred, in order to reduce antibiotic pressure in this extremely frequent entity [48]. In uncomplicated UTI, *E. coli* is the most common pathogen, typically being isolated from approximately 80% of outpatients with acute uncomplicated cystitis across the various regions of the world [49,50,51]. In clinical practice, urine culture is usually not performed in the setting of community-acquired, uncomplicated cystitis. Antibiotic therapy is therefore mostly empiric and more or less based upon knowledge of national or international surveillance studies. The local resistance levels of *E. coli* therefore determine the empiric antibiotic treatment. The range of pathogens associated with acute uncomplicated pyelonephritis is similar to that seen in acute uncomplicated cystitis [52]. The most recent surveillance study in Europe investigating uncomplicated cystitis is the Antimicrobial Resistance Epidemiology Survey on Cystitis (ARESC) project [53]. The results of the ARESC study showed that antibiotic substances classically used for the treatment of uncomplicated UTIs, such as cotrimoxazole, fluoroquinolones or aminopenicillins, lose their effectiveness due to increasing resistance. Therefore, ideal substances are those with high susceptibility rates, exclusively used for this indication, such as fosfomycin tromethamine, nitrofurantoin or pivmecillinam.

Finally, Slekoveck *et al*. [54] state that while long-term, prophylactic nitrofurantoin should be restricted, due to secondary effects, this antibiotic should retain its place in the therapeutic armamentarium for UTIs, especially in the face of rising multidrug-resistant Enterobacteriaceae. In their country of origin, prescribers largely replaced nitrofurantoin with fluoroquinolones due to the frequency of nitrofurantoin adverse reactions related to duration of therapy. This had a tremendous impact on increased fluoroquinolone consumption, which has been related to antimicrobial resistance.

## 7. Conclusions

Nitrofurantoin has been used for a long time, but the emergence of antibiotic resistance and the decline in newly developed antibiotics has increased interest in the treatment of bacterial UTI with this antibiotic. Pharmacology limitations, such as four doses per day needed, may be soon something of the past. Not only has resistance to nitrofurantoin remained virtually unchanged since its discovery but also its safety profile have made it the antimicrobial of choice in the prophylaxis and treatment of lower UTI in adults for many years. In this new microbiological era characterized by multi-drug resistant pathogens, nitrofurantoin’s role is crucial.
